# Early Impact of COVID-19 Pandemic on Burn Injuries, Admissions, and Care in a Statewide Burn Service

**DOI:** 10.3390/ebj3030039

**Published:** 2022-09-12

**Authors:** Lincoln M. Tracy, Cheng Hean Lo, Heather J. Cleland, Warwick J. Teague, Belinda J. Gabbe

**Affiliations:** 1 School of Public Health and Preventive Medicine, Monash University, Melbourne, VIC 3004, Australia; 2 Victorian Adult Burns Service, The Alfred, Melbourne, VIC 3004, Australia; 3 Department of Paediatric Surgery, The Royal Children’s Hospital, Melbourne, VIC 3052, Australia; 4 Burns Service, The Royal Children’s Hospital, Melbourne, VIC 3052, Australia; 5 Health Data Research UK, Swansea University Medical School, Swansea University, Singleton Park, Swansea, Wales SA2 8PP, UK

**Keywords:** burn, COVID-19, pandemic, Victoria

## Abstract

Anecdotal evidence from specialist burn clinicians suggested patient numbers and workloads increased during lockdown periods. This study aimed to describe the impact of the early COVID-19-related public health control measures (i.e., lockdowns) on burn injuries, hospital admissions, and care in a statewide burn service. We examined admissions data from The Victorian Adult Burns Service (located at the Alfred Hospital) and the Royal Children’s Hospital Burns Service—both of which contribute to the Burns Registry of Australia and New Zealand—during lockdown periods between March and October 2020, compared to the same periods in previous years. There were 714 patients admitted during the control period and 186 during the COVID-19 period. Burns sustained during COVID-19 lockdowns were larger in size. During COVID-19 lockdowns a greater proportion of patients were admitted to intensive care. Although the number of burn-related admissions did not increase during lockdowns, burn injuries that did occur were more severe (i.e., affected a greater percentage of body surface area). These more severe injuries placed an additional and significant burden on an already strained healthcare system. Future public health messaging should include prevention information to minimize the number of injuries occurring during lockdowns and other responses.

## 1. Introduction

The year 2020 saw the emergence of a global pandemic arising from a novel coronavirus disease (COVID-19). The first recorded case of the pandemic was recorded in Wuhan, China, in December 2019. In January 2020, the World Health Organization declared the outbreak a “public health emergency of international concern” before it was classified as a pandemic on 11 March 2020 [1]. Since the first COVID-19 case was identified there have been a further 250 million confirmed cases and more than five million deaths [2]. The true long-term health burden of COVID-19 remains to be seen.

Following the confirmation of the first Australian COVID-19 case on 25 January 2020 [3], State and Federal governments implemented a variety of public health control measures (i.e., lockdowns and other restrictions) in an attempt to limit transmission in March and April 2020. Changes in lockdown restrictions occurred throughout the year and were tailored to daily COVID-19 case numbers. Within Australia, residents of Victoria (particularly metropolitan Melbourne) lived under more severe restrictions for the longest duration relative to other States and Territories. Restrictions included school closures for extended periods, travel limits (i.e., residents were restricted to a five kilometre radius from their homes or usual places of residence), overnight curfews, and a “ring of steel” preventing movement into and out of metropolitan Melbourne. While these restrictions limited the transmission of COVID-19, they were not without unintended consequences. Many professional, societal, and behavioural aspects were required to pivot as parts of a COVID-19-affected health system and society, unfortunately leading to an increase in potentially risky behaviours due to changes in restrictions. Such behavioural considerations had acute relevance to the burn patient population and the specialist clinicians who treat them.

Numerous studies have explored how burn services have changed processes and protocols to manage COVID-19-positive burn patients or to minimise the risk of viral spread among patients and/or healthcare professionals [4,5,6,7,8,9,10,11,12]. Other studies have examined the effects of the pandemic on burns epidemiology in a variety of international settings (e.g., [13,14,15,16,17,18,19,20,21]). However, previous studies examining the impact of COVID-19 social restrictions across Australia and New Zealand have either focused on trauma more broadly or been limited just to a paediatric cohort [22,23,24,25,26,27,28]. There is yet to be a study examining the impact of the early stages of the COVID-19 pandemic on burn injury demographics, admissions, and management in the Australian state with the most severe and prolonged lockdowns in 2020 [29].

This cross-sectional registry-based study aimed to assess the impact of the COVID-19 pandemic and associated lockdown measures on burn injury admissions and care at specialist burn services in Victoria, Australia. Specifically, we wanted to determine whether the lockdown restrictions affecting metropolitan Melbourne changed the number, type, and management of burn injuries resulting in admissions to the two specialist Victorian burn services.

## 2. Materials and Methods

### 2.1. Setting and Data Source

The Victorian Adult Burns Service (VABS; located at the Alfred Hospital) and the Royal Children’s Hospital Burns Service are the statewide adult and paediatric burns services for the state of Victoria, Australia. These services manage almost all severely injured burns patients within the state. Both services contribute data to the Burns Registry of Australia and New Zealand (BRANZ), a bi-national clinical quality registry designed to capture epidemiological, quality of care, and in-hospital outcome data for adult and paediatric burn patients across Australian and New Zealand burn services. This study used non-probability (non-random) sampling to ensure patients included in the study were admitted to a specialist burn service and registered with the BRANZ (meaning their injuries were severe enough to require care at a specialist burn service) during the timeframe of interest (to compare differences before and during COVID-19 lockdown restrictions). Data for all acute admissions to the Alfred and Royal Children’s Hospitals recorded by the BRANZ between January 2016 and October 2020 were extracted. To be registered with the BRANZ, patients must be admitted to a specialist burn service within 28 days of the injury occurring. Transfers from other hospitals are included irrespective of the time from injury to admission. Admissions must last for more than 24 h, unless the patient undergoes a burn wound management procedure in theatre (in which case admissions lasting less than 24 h are included). All admissions resulting in an in-hospital death are included, regardless of the time elapsed since admission. Patients with an unknown date of injury were excluded. No other exclusion criteria were applied.

### 2.2. Data Management

Age at the time of injury was calculated using date of birth and date of injury data. In accordance with age-based definitions applicable to the Victorian State Trauma Registry, patients were further classified as paediatric (0–15 years) or adult (≥16 years) [30]. Residential postcodes were mapped to the Index of Relative Socio-economic Advantage and Disadvantage (IRSAD) [31] deciles, which were converted to quintiles.

Metropolitan Melbourne’s first lockdown lasted 43 days (31 March–12 May 2020) and the second lockdown lasted 111 days (9 July–27 October 2020). Acute burn admissions in 2020 were categorised according to whether lockdown restrictions were in place or not. Admissions were also grouped according to the year of admission; admissions in 2016 to 2019 were aggregated to represent the pre-COVID-19 period where appropriate. The time from injury to admission at a specialist burn service was calculated using date and time of injury and admission data. Referral sources to a specialist burn service were categorised as direct from the scene via ambulance, transferred from another (non-specialist) hospital, outpatients, or other source.

Primary burn injury causes were categorised as flame, scald, contact, chemical, and other cause. Activities at the time of injury were categorised as playing, near someone cooking and/or preparing food/drink, or other activity for paediatric patients, and cooking and/or preparing food/drink, leisure activity, work-related activity, household maintenance, sleeping/resting, or other activity for adult patients. Places at the time of injury were categorised as home (or usual place of residence) or other specified place. Injury intents were categorised as unintentional or other intent (including intentional self-harm and suspected assaults). Injury severity was primarily defined by the total body surface area (TBSA) burned; TBSA was reported as a continuous measure and categorised as follows: 0–4.9%, 5–9.9%, 10–19.9%, 20–49.9%, and ≥50%. Burn depth was dichotomised to identity patients with deep dermal and/or full thickness burns.

In-hospital management and outcome data (i.e., whether patients underwent a burn wound management procedure in theatre, whether they received a skin graft, whether they were admitted to the intensive care unit (ICU), how long they spent in ICU, and discharge disposition) were also extracted. Discharge dispositions were categorised as home/usual place of residence or other location. The acute admission LOS was calculated using date and time of admission and discharge data.

### 2.3. Statistical Analysis

Summary statistics were derived from extracted data. Frequencies and percentages described categorical variables. Continuous variables were described using mean and standard deviation or median and interquartile range (IQR) for normally and non-normally distributed variables. Data field entries coded as not stated, inadequately described, or not specified were considered missing and excluded from analysis. Cells in tables with low counts (i.e., less than five) were aggregated where possible. Data were analysed separately for paediatric and adult admissions where meaningful and possible. The mean of admissions and of injuries during the 2016–2019 period were calculated; a bootstrap of 10,000 samples defined the bias-corrected and accelerated 95% confidence intervals (CI) surrounding these mean values. Differences in demographic and injury event characteristics, management, and outcomes of patients were compared using independent samples t-tests, Mann–Whitney U-tests, and chi-square tests, as appropriate. A *p*-value < 0.05 was considered statistically significant. All data management and statistical analyses were undertaken in the R statistical environment version 4.0.3 [32] using the *tidyverse* [33], *lubridate* [34], *tableone* [35], *boot* [36,37], *RColorBrewer* [38], and *cowplot* [39] packages.

### 2.4. Ethics Approval

Ethics approval for this study was granted by The Alfred Hospital Ethics Committee (64/21) and the Royal Children’s Hospital Human Research Ethics Committee (QA/77922/RCHM-2021).

## 3. Results

**Figure 1:**
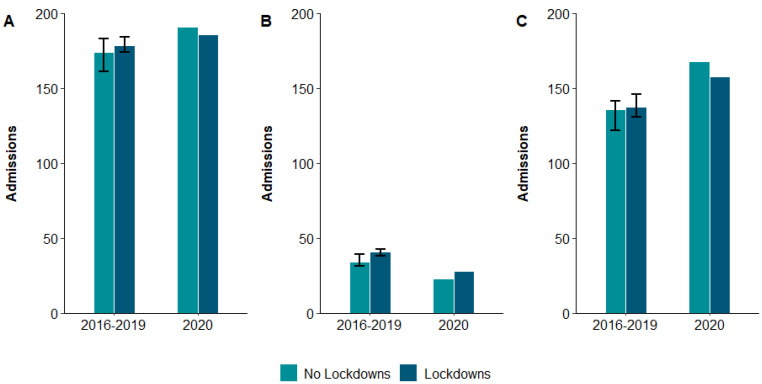
Admissions presenting to specialist Victorian burn services during and outside lockdown restriction periods in (**A**) all, (**B**) paediatric, and (**C**) adult patients, stratified by pre-COVID-19 (2016–2019) and COVID-19 (2020) epochs. The ‘lockdown’ bar in the 2016–2019 period represents the number of admissions that occurred during periods in these years when lockdown restrictions were in place in 2020 (i.e., 31 March–12 May and 9 July–27 October in 2016, 2017, 2018, and 2019). Error bars represent 95% CIs around bootstrapped means in the pre-COVID-19 epoch.

There were 714 patients admitted during the control period and 186 patients admitted during the COVID-19 period. There was no difference in the number of admissions during the COVID-19 period compared to the bootstrapped mean number of injuries during the pre-COVID-19 period for either the paediatric (*p* = 0.39) or adult (*p* = 0.59) cohorts (Figure 1).

Paediatric patients admitted during the Victorian lockdown periods in 2020 had a shorter median time to admission (median IQR 2(1–32) hours in 2020 lockdowns compared to 49 (3–281) hours pre-COVID-19; *p* < 0.001; see Table 1). A greater proportion of paediatric patients were taken directly from the scene of injury to a specialist burn service (54% in 2020 lockdowns versus 22% pre-COVID-19; *p* = 0.003). A greater proportion of paediatric patients injured during the Victorian lockdown periods in 2020 sustained scalds or flame burns compared to patients during the same periods in the preceding years, whereas the proportion of burns from all other causes decreased. Patients injured during COVID-19-related lockdowns in 2020 sustained more severe injuries compared to patients injured during the preceding years: a greater proportion sustained inhalation injuries, the median TBSA increased (from 6% (2–8%) pre-COVID-19 to 9% (5–16%) in 2020 lockdowns; *p* = 0.002), and there was a greater proportion of burns affecting ≥10% TBSA. Paediatric patients admitted during 2020 lockdown periods did not differ from patients admitted during non-lockdown periods with respect to age, gender, the activity when the injury occurred, socioeconomic status, injury intent, whether the injury occurred in the home, or the maximal recorded depth.

The proportion of paediatric patients injured during lockdown periods who underwent a burn wound management procedure in theatre did not differ between pre-COVID-19 and COVID-19 epochs, but the median time to first theatre was shorter in 2020 (1 (0–3) days) compared to the 2016–2019 pre-COVID-19 periods (7 (2–15) days; *p* < 0.001). The proportion of patients who received a skin graft did not differ, nor did the time to first skin grafting. A greater proportion of patients were admitted to the ICU (6% in the pre-COVID-19 period, 32% during COVID-19 lockdowns; *p* < 0.001), and although the increase in median ICU LOS did not reach statistical significance, these likely represent real increases in clinical and resource burdens. The median hospital LOS for paediatric burns admissions increased in 2020 (8 (2–16) days in 2020 versus 2 (1–5) days pre-COVID-19; *p* < 0.001), whereas the proportion of children discharged to a location other than their home or usual residence (e.g., inpatient rehabilitation services) decreased (from 96% in the pre-COVID-19 period to 82% during COVID-19 lockdowns; *p* = 0.03).

Adult patients admitted during Victorian lockdown periods in 2020 had a shorter median time to admission compared to patients admitted during the pre-COVID-19 period (9 (2–63) hours pre-COVID-19 versus 5 (2–21) hours in 2020; *p* = 0.03; see Table 2). A smaller proportion of adult patients injured in the COVID-19 epoch sustained their injuries while partaking in a leisure/sports activity or while working for income compared to patients injured in the pre-COVID-19 epoch. A greater proportion of adult burn injuries occurred in the home during the COVID-19 epoch (57% pre-COVID-19 versus 73% in 2020; *p* < 0.001). Patients injured during COVID-19-related lockdowns in 2020 sustained more severe injuries compared to patients injured during the preceding years: the median TBSA burned increased (from 4% (2–8%) pre-COVID-19 to 5% (2–10%) in 2020; *p* = 0.007), and there was a greater proportion of burns affecting ≥10% TBSA. There were no differences in the number of adult patients injured in the pre-COVID-19 and COVID-19 epochs with respect to age, gender, socioeconomic status, referral source, injury cause, intent, whether the patient sustained an inhalation injury, or the maximal recorded depth.

The proportion of adult patients injured during lockdown periods who underwent a burn wound management procedure in theatre did not differ between pre-COVID-19 and COVID-19 epochs, nor did the median time to first theatre. The proportion of patients who received a skin graft did not differ, nor did the time to first skin grafting. A greater proportion of patients were admitted to the ICU (15% pre-COVID-19 versus 26% in 2020; *p* = 0.002), and the median ICU LOS increased in 2020 compared to the pre-COVID-19 period (46 (23–169) hours pre-COVID-19 versus 145 (34–336) hours in 2020; *p* = 0.03). The proportion of patients who were discharged to their home or usual residence did not differ, and patients had an increased LOS in 2020 (7 (3–13) days pre-COVID-19 versus 9 (5–17) days in 2020; *p* < 0.001).

## 4. Discussion

Consistent with international reports from the United Kingdom [16], Canada [19], and the United States [21], we observed an increase in burn injury severity (i.e., percentageTBSA burned) during COVID-19 lockdown periods compared to pre-COVID-19 periods. These more severe injuries were associated with an increased workload for clinical staff, greater resource use, and reduced bed availability (especially in intensive care). This placed unnecessary burdens on an already strained healthcare system attempting to relieve pressure on acute and critical care services. The additional strain associated with COVID-19 patients has required hospitals to alter workflows and models of care, including temporarily closing outpatient clinics. The anticipated increased burden of burn injuries and associated admissions during lockdown periods meant that burn service activity levels were sustained (if not increased), while many other services were cut back. This placed significant staffing burden on these services, the scars of which remain for some clinicians today.

Anecdotal evidence suggests that the burns service at the Royal Children’s Hospital had a higher threshold for choosing inpatient/operative management over conservative/non-operative management for paediatric burns patients during the early stages of the pandemic, especially for borderline patients who may have been admitted for treatment prior to COVID-19. This may have reduced the number of paediatric admissions during lockdowns, which, in turn, may have led to adults accounting for a greater proportion of admissions. Therefore, an additional consequence is that paediatric patients who were admitted would have had more severe injuries, placing greater strain on inpatient and operative capacity. The lack of an observed change in paediatric admissions during lockdown periods is inconsistent with previous national and international reports of an increase in paediatric admissions for burns as a whole [27,40,41,42]. This lack of an observed change is also inconsistent with studies that focused on specific burn types, such as steam-related inhalation injuries and treadmill-related friction burns in paediatric patients [13,18]. Mixed reports of how the pandemic altered burn injury presentations/admissions may arise from differences in the restrictions specific to each jurisdiction or country.

It is unsurprising that a greater proportion of burns in adults during lockdown occurred at home, with stay-at-home orders and curfews being a key part of the Victorian government’s response to the pandemic. This observation is consistent with Farroha’s findings of an increase in burns occurring at home in the United Kingdom during lockdown periods [14]. It is somewhat surprising that an increase in the proportion of paediatric burns sustained at home did not also increase during the same periods. These results are inconsistent with other studies examining the effects of the pandemic on burns epidemiology [16,27]. However, as three quarters of paediatric burns in Australia and New Zealand occur in the home [43], a saturation effect may be occurring. The greater proportion of adult patients admitted during lockdown phases underlies the predominance of flame burns in the current study, as flame burns are the most common cause of burn injury in adult patients [43,44]. The increased prevalence of flame burns also contributed to the more severe injuries sustained during lockdowns: flame burns are typically larger compared to other burn causes and are more commonly associated with inhalation injury. Patients with more severe injuries are typically taken to hospital directly from the scene of injury, which also explains changes in admission source and median time to admission discussed earlier.

Differences in how patients were managed once admitted to hospital were observed between lockdown and non-lockdown periods of 2020, and can be explained by the increased injury severity seen in patients admitted during lockdown. Larger burns require surgical management more quickly due to the importance of early excision of injuries [45]. Patients with more severe injuries are also admitted to intensive care more frequently and remain longer as they require a higher level of care than patients who can be managed on the ward. Similarly, patients who survive with more severe injuries have a longer hospital LOS and are more commonly discharged to a rehabilitation hospital or other healthcare setting, rather than being discharged to their home or usual residence.

Although policy orders and directives encouraging (or mandating) individuals to stay at home were critical tools in limiting and preventing the transmission of COVID-19 throughout the community, current results indicate that future responses such as these also need to include an injury prevention component. The proportional increase in unintentional injuries occurring in the home during these restriction periods [46] suggests that a “stay at home, but stay safe while at home” line of public health messaging may be a useful prevention tool to consider in future.

This study was limited by only focusing on admissions to specialist burn services at two hospitals. Examining data from all Victorian hospitals may provide a more complete picture of the impact of burn injuries during COVID-19 lockdowns [46]. However, no other data source has as fine a level of detail regarding burn injury characteristics as the BRANZ. Further research is required to examine the effects of the latter stages of the pandemic (i.e., additional lockdown periods in 2021) to fully understand the impact of COVID-19 lockdowns on burn injuries as well as their admissions and care.

## 5. Conclusions

These findings underline the importance of maintaining ever-relevant injury prevention information (i.e., how to keep safe in and around the home) coupled with relevant COVID-19 public health messaging when announcing restrictions such as lockdowns or stay-at-home orders.

## Figures and Tables

**Table 1:** Demographic, injury, and outcome data for paediatric patients admitted to a specialist Victorian burn service during lockdown periods, 2016–2020.

	Pre-COVID-19 Period (2016–2019)	COVID-19 Lockdowns (2020)	*p*
**Number of patients**	155	28	
**Age, median (IQR) years**	2 (1–7)	2 (1–11)	0.95
**Male**	99 (64%)	16 (57%)	0.64
**Primary cause**			0.005
Scald	83 (54%)	17 (61%)	
Flame	26 (17%)	10 (36%)	
Other cause	45 (29%)	<5	
**Activity when injury occurred**			0.56
Near person preparing food	46 (32%)	11 (41%)	
Playing	58 (41%)	11 (41%)	
Other activity	39 (27%)	5 (18%)	
**IRSAD quintile**			0.45
1 (most disadvantaged)	40 (26%)		
2	36 (23%)		
3	23 (15%)		
4	27 (18%)		
5 (least disadvantaged)	27 (18%)		
**Injury occurred in home**	118 (78%)	22 (79%)	0.99
**Unintentional injury**	152 (98%)	27 (96%)	0.99
**Time to admission, median (IQR) hours**	49 (3–281)	2 (1–32)	<0.001
**Admission source**			0.003
Scene via ambulance	35 (22%)	15 (54%)	
Other hospital	46 (30%)	5 (18%)	
Outpatient department	60 (39%)	<5	
Other source	14 (9%)	<5	
**TBSA burned, median (IQR)%**	6 (2–8)	9 (5–16)	0.002
**TBSA group**			0.003
0–4.9%	68 (44%)	7 (25%)	
5–9.9%	56 (37%)	9 (33%)	
10–19.9%	23 (15%)	6 (21%)	
≥20%	6 (4%)	6 (21%)	
**Deep dermal or FT burn**	78 (70%)	20 (70%)	0.79
**Inhalation injury**	<5	5 (18%)	<0.001
**Burn wound management in theatre**	114 (74%)	21 (75%)	0.99
**Time to first procedure, median (IQR) days**	7 (2–15)	1 (0–3)	<0.001
**Received skin graft**	67 (59%)	7 (33%)	0.06
**Time to first skin graft, median (IQR) days**	15 (9–18)	16 (13–17)	0.58
**Admitted to ICU**	9 (6%)	9 (32%)	<0.001
**ICU LOS, median (IQR) hours**	111 (89–231)	589 (130–1080)	0.12
**Discharged to home/usual residence**	148 (96%)	23 (82%)	0.03
**Hospital LOS, median (IQR) days)**	2 (1–5)	8 (2–16)	<0.001

Data are presented as frequency (percentage) unless otherwise specified. Excludes missing data. FT = full thickness; ICU = intensive care unit; IQR = interquartile range; IRSAD = Index of Relative Social Advantage and Disadvantage; LOS = length of stay; TBSA = total body surface area.

**Table 2:** Demographic, injury, and outcome data for adult patients admitted to a specialist Victorian burn service during lockdown periods, 2016–2020.

	Pre-COVID-19 Period (2016–2019)	COVID-19 Lockdowns (2020)	*p*
**Number of patients**	540	160	
**Age, median (IQR) years**	41 (27–57)	40 (27–54)	0.64
**Male**	399 (74%)	122 (76%)	0.62
**Primary cause**			0.005
Scald	126 (23%)	39 (25%)	
Flame	320 (60%)	102 (64%)	
Other cause	90 (17%)	18 (11%)	
**Activity when injury occurred**			0.003
Cooking and/or preparing food/drink	89 (17%)	28 (19%)	
Leisure or sports activity	86 (17%)	17 (11%)	
Other household duties/maintenance	71 (14%)	20 (14%)	
Sleeping or resting	60 (11%)	10 (7%)	
Other activity	131 (25%)	55 (37%)	
**IRSAD quintile**			0.08
1 (most disadvantaged)	88 (17%)	26 (17%)	
2	97 (18%)	37 (24%)	
3	118 (23%)	24 (15%)	
4	103 (20%)	41 (27%)	
5 (least disadvantaged)	114 (22%)	27 (17%)	
**Injury occurred in home**	290 (57%)	110 (73%)	<0.001
**Unintentional injury**	511 (95%)	148 (93%)	0.42
**Time to admission, median (IQR) hours**	9 (2–63)	5 (2–21)	0.03
**Admission source**			0.43
Scene via ambulance	198 (37%)	62 (39%)	
Other hospital	250 (46%)	75 (47%)	
Outpatient department	21 (4%)	< 5	
Other source	71 (13%)	21 (13%)	
**TBSA burned, median (IQR)%**	4 (2–8)	5 (2–10)	0.007
**TBSA group**			0.03
0–4.9%	297 (55%)	73 (46%)	
5–9.9%	131 (24%)	37 (23%)	
10–19.9%	64 (12%)	33 (21%)	
≥20%	46 (9%)	15 (10%)	
**Deep dermal or FT burn**	297 (56%)	89 (56%)	0.95
**Inhalation injury**	24 (5%)	14 (9%)	0.05
**Burn wound management in theatre**	387 (72%)	122 (76%)	0.23
**Time to first procedure, median (IQR) days**	3 (1–6)	2 (1–5)	0.05
**Received skin graft**	299 (78%)	96 (79%)	0.83
**Time to first skin graft, median (IQR) days**	5 (3–8)	4 (3–8)	0.56
**Admitted to ICU**	83 (15%)	42 (26%)	0.002
**ICU LOS, median (IQR) hours**	46 (23–169)	145 (34–336)	0.03
**Discharged to home/usual residence**	290 (54%)	85 (53%)	0.90
**Hospital LOS, median (IQR) days)**	7 (3–13)	9 (5–17)	<0.001

Data are presented as frequency (percentage) unless otherwise specified. Excludes missing data. FT = full thickness; ICU = intensive care unit; IQR = interquartile range; IRSAD = Index of Relative Social Advantage and Disadvantage; LOS = length of stay; TBSA = total body surface area.

## Data Availability

Data presented in this study are not publically available due to ethical restrictions.
